# Editorial Comment on “Predictive value of haematologic parameters and HALP score for testicular viability in adults with testicular torsion: A multicentric study”

**DOI:** 10.1111/iju.15669

**Published:** 2025-01-10

**Authors:** Roy Mano

**Affiliations:** ^1^ Department of Urology Tel Aviv Sourasky Medical Center Tel Aviv‐Yafo Israel; ^2^ Faculty of Medical & Health Sciences Tel Aviv University Tel Aviv‐Yafo Israel

Testicular torsion is associated with an orchiectomy rate of 39% and a late testicular loss rate of approximately 50% in the pediatric and adolescent population.^1^ Previous studies report a consistent association between symptom duration, degree of torsion, and sonographic features before scrotal exploration and testicular salvage after torsion.^1^, ^2^ Biomarkers based on the results of a complete blood count are appealing as predictors of outcome since they are readily available and have been previously associated with various inflammatory conditions. However, when evaluating the association between the outcome of scrotal exploration and blood‐based biomarkers including immune cell counts, neutrophil‐lymphocyte‐ratio, platelet‐lymphocyte‐ratio, and C‐reactive protein, findings are inconsistent.^3^

In the current study, Yilmaz et al. evaluated hematological variables, and the hemoglobin, albumin, lymphocyte, and platelet (HALP) score as predictors of testicular viability in adults with testicular torsion.^4^ The HALP score was previously associated with vascular disorders and the vitality of free microvascular flaps and may therefore be a potential biomarker for testicular salvageability. The authors report that patients who underwent orchiectomy had significantly lower HALP scores (43.7 ± 33.9 vs. 56.6 ± 44.3, adjusted *p*‐value = 0.03), higher platelet to lymphocyte ratio, higher degree of torsion, higher MCV, longer duration of torsion and heterogenous echotexture on sonography. However, multivariable logistic regression analysis revealed that only heterogeneous echotexture (OR = 14.3, 95% CI = 2.4–85.5, *p* = 0.004) and duration of torsion (OR = 1.1, 95% CI = 1–1.1, *p* < 0.001) were significant predictors for orchiectomy on scrotal exploration; thus, the authors did not find an association between the HALP score and additional hematological markers and the outcome of testicular torsion.^4^ The authors acknowledge the limitations of the study including its retrospective nature, small cohort size, and lack of long‐term follow‐up. Despite these limitations, preoperative sonographic echotexture and duration of symptoms were found to be predictors of outcome at scrotal exploration consistent with previous studies.

Gross testicular heterogeneity on preoperative ultrasonography was repeatedly associated with testicular loss following torsion. In an attempt to provide an objective measure of sonographic heterogeneity, Samson et al. analyzed ultrasound images of the testes with a computer software and compared the heterogeneity index of the affected and contralateral testis providing an objective variable able to determine the viability of a torsed testis with a sensitivity, specificity, positive predictive value and negative predictive value of 100%, 94.5%, 86%, and 100%, respectively.^5^ While all cases of torsion should be treated in an urgent manner, preoperative ultrasonography may be used to predict testicular viability and assist in patient counseling regarding the expected outcome of scrotal exploration. Furthermore, if these findings are supported by large prospective studies, they may possibly justify delaying scrotal exploration for non‐viable testes in the few cases with substantial confounding surgical risks.

## CONFLICT OF INTEREST STATEMENT

The authors declare no conflicts of interest.

## FUNDING INFORMATION

No financial support was obtained for the current work.
